# Effect of Postbiotic Supplementation on Nutrient Digestibility and Milk Yield during the Transition Period in Dairy Cows

**DOI:** 10.3390/ani14162359

**Published:** 2024-08-15

**Authors:** Fernando Vicente, María Campo-Celada, Mario Menéndez-Miranda, Jairo García-Rodríguez, Adela Martínez-Fernández

**Affiliations:** Servicio Regional de Investigación y Desarrollo Agroalimentario (SERIDA), Carretera AS-267, PK. 19, 33300 Villaviciosa, Asturias, Spain; mcampo@serida.org (M.C.-C.); mmiranda@serida.org (M.M.-M.); jairogr@serida.org (J.G.-R.); admartinez@serida.org (A.M.-F.)

**Keywords:** postbiotic, dairy cow, voluntary intake, digestibility, immunoglobulin, milk yield

## Abstract

**Simple Summary:**

Postbiotics are non-viable probiotic microorganisms, with or without their metabolites, that provide a health benefit to the host. They prevent the colonisation of pathogens by improving the gut environment for beneficial commensal bacteria. This effect reduces the incidence of digestive disorders in calves, which enhances animal growth and boosts the immune system and general health. The aim of this work was to evaluate the addition of postbiotics to dairy cow rations during the transition period on nutrient digestibility and milk productivity. The effects of two postbiotics from calving to two months of lactation were evaluated in twelve Friesian cows. The results suggest that postbiotic supplementation in late gestation and early lactation increases the voluntary intake of dry matter, with higher apparent total tract digestibility of nutrients. The inclusion of postbiotics in the dairy cow diet increases colostral immunoglobulin concentration and milk production with high fat and protein yields.

**Abstract:**

The metabolites secreted by probiotics or released after their lysis are called postbiotics. They provide physiological benefits to the host, preventing the colonisation of pathogens by improving the intestinal environment for beneficial commensal bacteria, which reduces the incidence of digestive disorders and improves the immune system. The aim of this work was to evaluate the addition of postbiotics to dairy cow rations during the transition period on nutrient digestibility, composition, and milk yield. The effects of two postbiotics were evaluated in twelve Friesian cows from 30 days before calving to two months of lactation. The animals were randomly allocated to two treatment groups: control (CT) and supplemented with postbiotics (PC and PR). Feeding was ad libitum with 60/40 of forage/concentrate ratio on dry matter basis. Daily feed intake and milk production were recorded individually throughout the study. Two digestibility balances were performed, one before parturition and one after parturition. Colostrum was sampled at first milking and milk was sampled weekly. Data were analysed using a mixed mode in R software 4.4.1. The results suggest that postbiotic supplementation in late gestation and early lactation increase the voluntary intake of dry matter, especially in the PR treatment, with higher apparent total tract digestibility of dry matter, organic matter and neutral detergent fibre. Both treatments including postbiotics induced an increase in colostral immunoglobulin concentration. Milk production of cows receiving the PC treatment was the highest, with high fat and protein yields and a higher persistence of the production curve throughout the lactation.

## 1. Introduction

Ruminants have the ability to convert fibre-rich forage, which is indigestible to monogastrics, into a high-quality nutrient source. In grazing and mixed systems, the intake of ruminants relies mainly (about 90%) on forage, i.e., grass, silage and crop residues [1]. However, the high nutritional requirements of dairy cows during late gestation and early lactation fall within a period of low voluntary intake capacity. Thus, the supplementation with concentrate or improving forage digestibility is required to overcome this energy gap. Dairy cows are often fed energy-dense diets with high levels of starchy concentrates to meet all the animal’s requirements. However, the rapid fermentation of starch by the rumen microbiome can reduce rumen pH and increase the risk of metabolic disorders [2]. Therefore, it is considered essential to improve the fibre digestibility of forages to increase nutrient utilisation during the transition period of cows when their requirements exceed their voluntary intake of forages.

The use of bacterial and yeast fermentation products as feed additives has been proposed to improve ruminal fermentation [3]. For example, yeast products containing *Saccharomyces* and *Aspergillus* can improve ruminal fermentation by increasing feed digestibility, whilst lactic-acid-producing bacteria can modulate the microbial balance in the gastrointestinal tract and improve feed efficiency. The beneficial health effects of probiotics have led to increasing scientific interest in the use of products derived from microbial metabolism. Probiotics are non-pathogenic living organisms which remain active in the gut and modify the host’s digestive microbiota when they are added to the diet in an adequate concentration and quantity [4]. However, the use of probiotics requires a cautious handling since they are living organisms which could lose their beneficial activity if they suffer adverse environmental conditions. They also carry potential risks such as infection and transfer of antibiotic resistance to pathogenic microbes, as well as excessive immune response, and even errors in dosage, formulation, and route of administration [5]. The metabolites secreted by probiotics or released after their lysis also provide physiological benefits to the host and would avoid the risks associated with the administration of live bacteria. These soluble factors have been proposed as alternatives to the use of probiotics, termed postbiotics [6], which exert a probiotic effect in the absence of live microbial cells [7]. Although postbiotics do not contain live bacteria, the presence of metabolites from probiotic bacteria allows them to benefit host health through mechanisms similar to those of probiotics [8]. Postbiotics have a well-defined chemical structure (such as enzymes, peptides, teichoic acids, peptidoglycan-derived muropeptides, polysaccharides, cell surface proteins, and organic acids), safe dosage parameters and a long shelf life [9]. Postbiotics prevent the colonisation of pathogens by improving the intestinal environment for beneficial commensal bacteria [10]. The organic acids and bacteriocins in postbiotics reduce the pH of the gut, inhibiting the proliferation of opportunistic pathogens, and promoting the growth of beneficial bacteria that modulate the microbial balance and help maintain gut health [3]. In vivo studies suggest that they act as a biofilm that coats the intestinal surface facing the gut lumen, preventing the adhesion of pathogens [11]. Hence, postbiotics reduce the incidence of digestive disorders and improve the immune system [12]. In addition, postbiotics have shown to cause an increase in fibre digestion in in vitro studies [13], which may help to improve the ruminal fermentation. Also, postbiotic supplementation in lactating goats improved fibre digestibility and energy efficiency for milk production [14].

Based on these findings, it was hypothesised that the addition of postbiotics to dairy cows’ rations during the transition period could improve nutrient digestibility and increase productivity and milk yield. Therefore, the present study was designed to test the effects of including postbiotics in the diet of dairy cows on prepartum and postpartum feed digestibility, early lactation milk yield, and the composition of milk.

## 2. Materials and Methods

### 2.1. Ethics Statement

The trial was carried out at the SERIDA Demo-Farm located in Villaviciosa, Asturias, Spain (43°28′20.3′′ N 5°26′10.0′′ W) with the approval of the Regional Office for Animal Health and Animal Production (PROAE 18/2022), in accordance with the guidelines set out in the European Community Council Directive 2010/63/EU on the protection of animals used for scientific purposes.

### 2.2. Animals and Diets

Twelve Friesian dairy cows were selected for the experiment. The cows were six first-calving heifers and six second-calving cows, with a body weight of 621 kg ± 98.6 (mean ± standard deviation) and a body condition score (BCS) of 2.96 ± 0.235 (scale from 1 to 5). The pregnancies of all animals were synchronised so that calving occurred in early autumn 2022, with a difference of 37 days between the first and last calving. Feeding was ad libitum for both pre- and postpartum, with total mixed rations (TMR) formulated according to NRC [15] with a 60/40 forage/concentrate ratio on a dry matter basis. In addition, all animals received an additional concentrate twice daily as an energy supplement: 3 kg as fed daily before calving and adjusted to the production level after calving: 3 kg as fed/day as a base dose that was increased at a rate of 0.2 kg as fed per kg of milk produced above the average milk yield of the herd, up to a maximum of 6 kg/day. Fresh water was continuously available for animals. The ingredients and chemical composition of the diets and concentrates are listed in Table 1.

### 2.3. Experimental Design and Measurements

The feeding trial was arranged in a longitudinal, randomised block design with the animals allocated to three feeding groups (n = 4 per treatment) starting 45 days before the expected calving date and continuing for 60 days after calving. Climate variables such as mean, minimum, and maximum temperatures, rainfall, relative humidity, and mean and maximum temperature–humidity index were recorded daily at the meteorological station located at the SERIDA Demo-Farm. To minimise the differences among groups, cows were grouped by number of lactations (two first and two second-lactation animals in each group), body weight and body condition score. Weight and body condition scores were recorded fortnightly. Two postbiotics supplied by Pentabiol S.L., (Noáin, Navarra, Spain) were tested. The postbiotics were included in the concentrate when it was manufactured. The postbiotics were prepared using wheat bran as excipient so that in the manufacture of the concentrate the appropriate proportion of unsupplemented wheat bran was replaced by that supplemented with each postbiotic. The supplementation per cow of both postbiotics was 8 g/d before calving and 15 g/d after calving. Each group of animals was assigned to an experimental treatment: control (CT), Probisan-Ruminants-C^®^ (PC), and Probisan-Ruminants^®^ (PR). Daily TMR intake was recorded individually throughout the trial by a computerised electronic system integrated with a weighing pen, while daily concentrate intake was recorded by an automatic feeder attached to the milking system (DeLavalVMS^TM^, DeLaval International AB, Tumba, Sweden). The TMR and the additional concentrate were sampled fortnightly throughout the experiment. A sample of 40 mL of colostrum was collected at first milking. Samples were kept refrigerated until analysis. Milk yield was recorded at both milkings at 07:00 h and 19:00 h and sampled weekly. Morning and evening milk samples from each cow were mixed according to milk yield to produce a representative sample of 40 mL per day and cow, to which 0.13 mL of azidiol was added. Samples were kept refrigerated until analysis.

Two digestibility balances were made 15 days before expected calving and 30 days postpartum, respectively. Two cows from each treatment, one from the first and one from the second calving, were randomly selected and placed in individual metabolic cages for 7 days to establish pre- and postpartum digestibility balances. The same cows were used in both digestibility balances. Animals were housed in metabolism cages throughout the digestibility balance to measure daily feed intake, milk yield, and faecal and urinary output. After two days of acclimatisation to the cages and for a period of 5 days, daily records of feed offered and refused, total faeces and urine output, and milk yield in the postpartum balance, were made for each cow to calculate the nutrient balance. An aliquot of 2% of the total daily faecal output was collected from the collector box to provide a composite sample for each animal and period, and stored at –20 °C until analysis. Total daily urine was collected through an external polyethylene vinyl acetate separator attached to the vulva with a biocompatible adhesive in plastic drums containing 1 L of a sulphuric acid solution (10% *v*/*v*) to prevent microbial degradation and loss of volatile ammonium. After recording the weight and the specific gravity, a sample of 1% of the daily urine production was collected and pooled on an animal basis to provide a composite sample for each animal and period, which was at –20 °C until analysis. Cows were milked twice daily at 07:00 h and 19:00 h. Milk was collected from each animal on the second and fourth sampling days and milk samples were pooled and stored until analysis as described above.

### 2.4. Chemical Analyses

TMR samples were dried at 60 °C for 24 h and ground through a 0.75 mm sieve, and the additional concentrate samples were ground to 1 mm. Both samples were analysed by NIRS (Foss NIRSystem 5000, FOSS, Silver Spring, MD, USA) for dry matter (DM), ash, crude protein (CP), crude fibre (CF), ether extract (EE), starch, neutral detergent fibre (NDF), and acid detergent fibre (ADF). Ammonia N content of fresh faeces and urine was determined by the Kjeldahl method (Kjeltec 8400, FOSS Tecator, Hillerød, Denmark) after the alkalinisation of the samples with magnesium oxide (10% *v*/*v*). The dry matter of faeces was determined by drying samples to a constant weight at 60 °C, and the ash by combustion at 550 °C (TGA-601 Thermogravimetric analyser, Leco Corporation, St. Joseph, MI, USA). The concentration of NDF in faeces was determined by the Van Soest method [16]. The total N content of faeces and urine was determined by the Kjeldahl method, using Cu as the catalyst. Immunoglobulin of colostrum samples was analysed immediately after collection using an optical Brix refractometer (LLG-uniREFRACTO 5 pro, Lab Logistics Group GmbH, Meckenheim, Germany), then 0.13 mL of azidiol was added to 40 mL of colostrum and kept refrigerated until the fat and protein contents were analysed by the gravimetric method [17] and Kjeldahl method [18], respectively. Milk samples were analysed for fat, protein, lactose, solids-not-fat, and urea content with a Fourier-transform infrared spectrophotometer (MilkoScan FT 6000, FOSS Tecator, Hillerød, Denmark).

### 2.5. Calculations and Statistical Analysis

Organic matter (OM) and nitrogen-free extract (NFE) were calculated as:

OM (%) = 100 − Ash (%)(1)

NFE (%) = OM (%) − CP (%) − CF (%) − EE (%)(2)

The percentage of immediate principles is expressed on a dry matter basis.

The net energy for lactation (NE_milk_) of total mixed rations and additional concentrate was related to the concentration of metabolisable energy (ME) in the feedstuff using the NRC equation [15]:

NE_milk_ (Mcal/kg DM) = 0.1569 × ME (MJ/kg DM) − 0.07 (3)

where ME was estimated as the average of the metabolisable energy calculated according to MAFF [19] and ADAS [20]:

ME_MAFF_ = 10 × [(0.012 × CP (%)) + (0.031 × EE (%)) + (0.005 × CF (%)) + (0.014 × NFE (%))] (4)

ME_ADAS_ = 11.78 + (0.0654 × CP (%)) + (0.0665 × EE2 (%)) + (0.0414 × EE (%) × FB (%) − (0.018 × Ash (%)) (5)

The percentage of immediate principles is expressed on a dry matter basis.

Apparent digestibility coefficients (ADC) of nutrients (on a dry matter basis) were calculated for each animal as:

ADC (%) = [(nutrient intake − faecal excretion) ÷ nutrient intake] × 100(6)

The concentration of immunoglobulin G (IgG) in colostrum was estimated according to the equation [21]:

IgG (g/L) = −61.896 + 5.666 × °Brix(7)

Nitrogen conversion efficiency (NCE) was calculated as the ratio of daily milk protein yield to daily nitrogen intake.

Fat and protein corrected milk (FPCM) yield was calculated according to IDF [22]:

FPCM (kg/d) = kg of milk × [(0.1226 × milk fat (%)) + (0.0776 × milk protein (%)) + 0.2534(8)

Statistical analyses were performed using the R statistical software 4.4.1 [23]. For each variable tested, the mixed model included the fixed effects of treatment and trial day, and their interaction. Cow and number of lactations were considered as random effects. ‘Day’ was considered as a repeated measure with ‘cow’ as the subject. Multiple comparisons among treatments were evaluated using Duncan’s test. The significance level was set at *p* ≤ 0.05 and a trend was considered when 0.05 < *p* ≤ 0.10.

## 3. Results

There were no periods of heat stress in the animals according to the meteorological data provided during the trial. Cows experienced physiological weight gain at the end of gestation, as well as the typical decline in body weight and body condition during the transition to lactation (*p* < 0.05), although no differences among treatments were observed in these parameters. On average, cows started the trial with a body weight of 621 kg ± 98.6 and a body condition score of 2.96 ± 0.235, and reached calving with a body weight of 679 kg ± 85.5 and a body condition score of 3.21 ± 0.144. After 60 days in milk, the cows lost an average of 96 kg of body weight, whilst BCS decreased by 0.7 points.

Cows calved normally without intervention, including one cow that gave birth to twins. All calves born were normal and healthy, with an average birth weight of 41.5 kg ± 4.24, with no differences among the experimental treatments.

Nutrient intake and digestibility before calving are shown in Table 2. Dry matter and organic matter intakes were higher in the PR treatment than in the CT treatment (*p* < 0.05), whereas the PC treatment showed intermediate values with no differences between the other treatments. Crude protein and neutral detergent fibre intakes were higher in both treatments supplemented with postbiotics than in the CT treatment (*p* < 0.05). The nutrient balance showed that the digestibility of dry matter and organic matter tended to be higher in both supplemented treatments than in the control treatment (*p* < 0.1). As a result, the intake of digestible organic matter was higher in both supplemented treatments compared to the control (5.84 kg/day vs. 6.38 and 6.58 kg/day for CT, PC, and PR, respectively, *p* < 0.05). Crude protein digestibility was higher in the PC treatment compared to the control (*p* < 0.05), while the PR treatment showed intermediate values. Thus, the intake of digestible crude protein was higher in both postbiotic treatments compared to the control (0.87 kg/day vs. 1.08 and 0.99 kg/day for CT, PC, and PR, respectively, *p* < 0.05). However, no differences in nitrogen balance were observed among treatments.

Table 3 shows nutrient intake and digestibility after calving. The intakes of dry matter, organic matter, and neutral detergent fibre were higher in the PR treatment than in the CT and PC treatments (*p* < 0.05). Crude protein intake was higher in the CT and PR treatments, with no differences between them. However, the PC treatment had a lower crude protein intake than the other treatments (*p* < 0.05).

**Figure 1:**
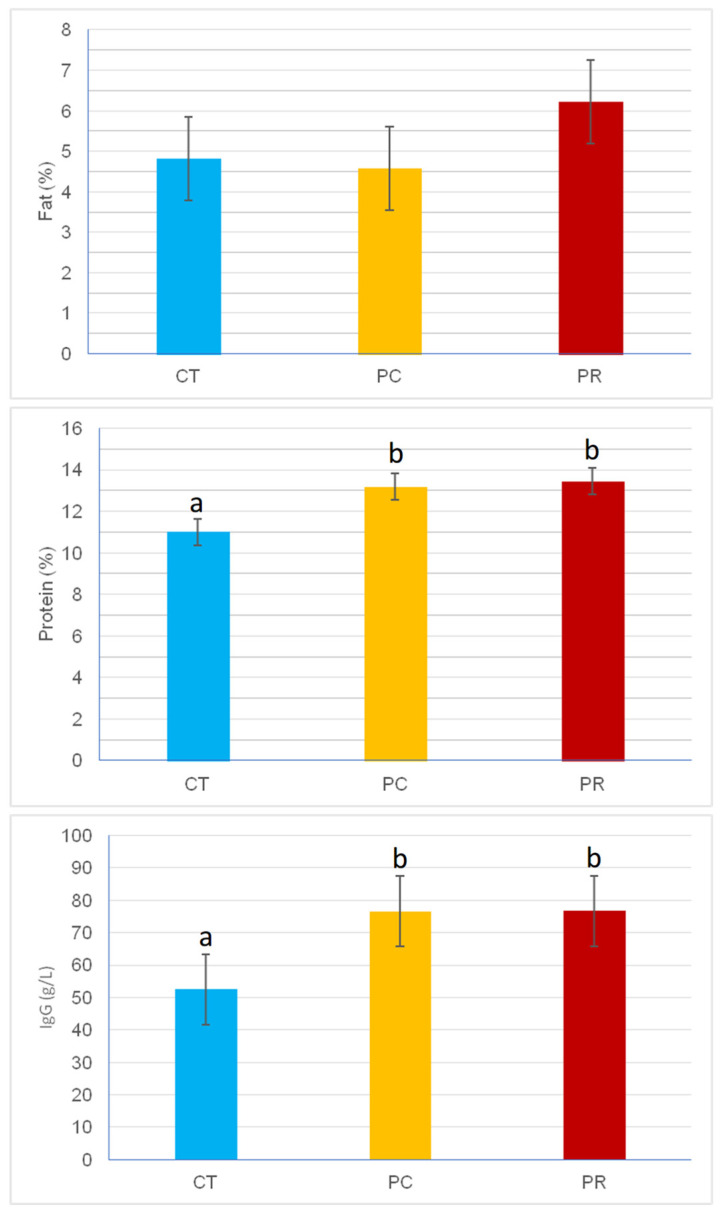
Concentration of fat (%), protein (%), and immunoglobulin G (g/L) in colostrum from cows in each of the experimental treatments. CT: control; PC: Probisan-Ruminants-C^®^; PR: Probisan-Ruminants^®^; different letters indicate statistical differences between treatments.

The composition of colostrum is shown in Figure 1. The colostral protein concentration was significantly higher in the supplemented treatments than in the control (11.00% vs. 13.44% and 13.18% for CT, PC, and PR, respectively, *p* < 0.05), as well as the levels of immunoglobulins (52.46 g/L vs. 76.65 g/L and 76.61 g/L for CT, PC, and PR, respectively, *p* < 0.01). In contrast, no differences in colostral fat concentrations were observed among treatments.

Table 4 shows the results of milk production and composition in the experimental treatments. The highest milk production was observed at the PC treatment, which was statistically different (*p* < 0.05) from the PR and the non-supplemented treatments.

**Table 4:** Milk production and milk composition in each experimental treatment.

	CT	PC	PR	rsd	*p*
Milk (kg/d)	29.61 ^b^	33.73 ^a^	30.06 ^b^	6.908	0.044
Fat (%)	4.03	4.19	3.80	0.668	0.068
Protein (%)	3.10	3.22	3.27	0.307	0.083
Solids-not-fat (%)	8.73 ^b^	8.68 ^b^	8.99 ^a^	0.413	0.007
Lactose (%)	4.83	4.79	5.08	0.648	0.148
Urea (mg/kg)	223	210	229	48.3	0.296
Milk performance					
FPCM (kg/d)	29.26 ^b^	34.30 ^a^	29.12 ^b^	7.046	0.004
kg fat/d	1.16 ^b^	1.42 ^a^	1.13 ^b^	0.336	0.002
kg protein/d	0.92 ^b^	1.08 ^a^	0.98 ^b^	0.211	0.014
Nitrogen efficiency (%)	34.16	39.93	36.68	9.546	0.078

CT: control; PC: Probisan-Ruminants-C^®^; PR: Probisan-Ruminants^®^; rsd: residual standard deviation; *p*: significance, different letters in the same item indicate statistical differences between treatments.

**Figure 2:**
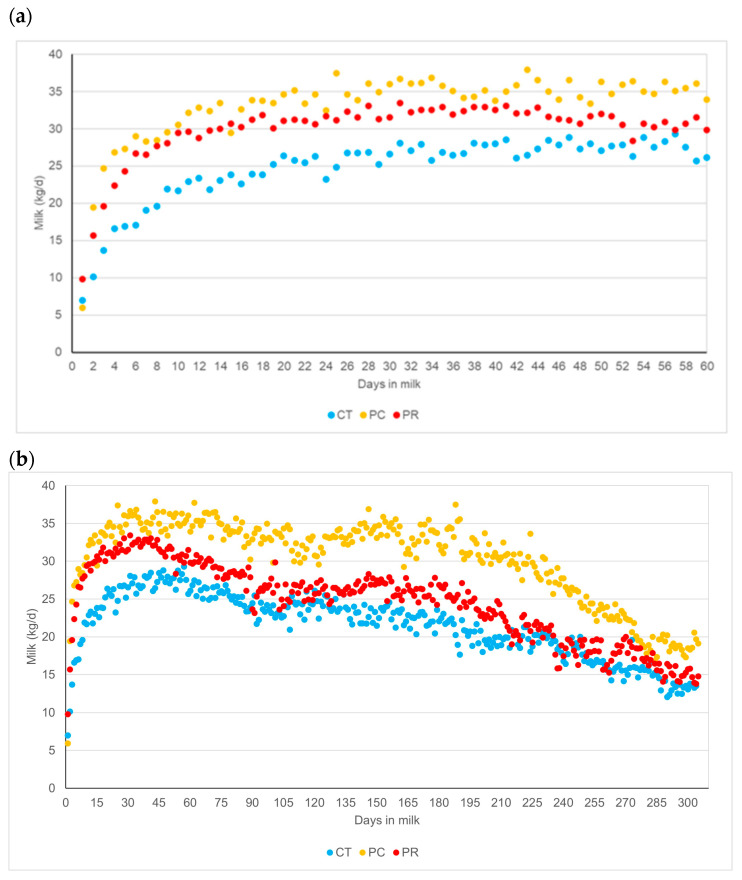
Milk production with the experimental diets during the experimental postpartum period (**a**) and throughout the lactation after the end of the study when all animals were fed with the same ration without postbiotic supplementation (**b**). CT: control; PC: Probisan-Ruminants-C^®^; PR: Probisan-Ruminants^®^.

Figure 2 shows the milk production for each of the experimental treatments up to day 60 postpartum of the experiment (a) and the entire lactation curve including data after the end of the study when all animals were fed with the same ration without postbiotic supplementation (b). Non-supplemented cows always had lower milk production than those receiving postbiotics. Both PC and PR treatments had similar milk yields up to the peak of lactation (around 35 days in milk). Thereafter, milk production decreased faster in the PR treatment than in the PC treatment, which was the key to the statistical differences between the postbiotic-supplemented treatments. Consequently, a higher persistence of the lactation curve was observed in the PC treatment than in the PR treatment throughout the lactation with a higher total milk yield. Milk protein concentration tended to be higher in the PC and PR treatments than in the CT treatment (*p* < 0.1). The highest fat concentration occurred in the PC treatment, with a trend towards higher concentrations than in the CT and PR treatments (*p* < 0.1). Solids-not-fat were higher in milk from cows supplemented with PR than in the PC and CT treatments (*p* < 0.05). When the milk was corrected for fat and protein concentration, the highest milk yield was also observed in the PC treatment and was statistically different (*p* < 0.05) to the PR and the CT treatments. Consequently, milk performance was better in the PC treatment, with higher protein and fat yields, than in the PR and the CT treatments (*p* < 0.05). The efficiency of dietary nitrogen utilisation also tended (*p* < 0.1) to be better in cows supplemented with postbiotic PC.

## 4. Discussion

The effect of postbiotics on voluntary intake in dairy cows varies between studies. While some authors observed no effect of postbiotic supplementation during the peripartum period [24], others reported an increase in voluntary intake immediately before and after calving [25] or immediately after calving [26]. These authors speculate that postbiotic supplementation reduces inflammation and stress around parturition, which increases voluntary intake immediately afterwards. This point is supported by the fact that serum cortisol, a biomarker of stress and pain [27], decreases in the days immediately following parturition [28], suggesting that *Saccharomyces cerevisiae* fermentation product supplementation may reduce parturition stress. Previous studies have suggested that yeast-derived products can be used in stressed animals as they may also have increased nutritional requirements during these periods [29]. The different results reported in the literature have been attributed to high inter-animal variability [30], as they appear to depend on breed, animal type, age, and physiological phase. For example, Jersey-Holstein cross calves supplemented with postbiotics similar to those used in our study had increased dry matter intake from weaning to 10 weeks of age, while no effect of supplementation was observed after 10 weeks of age [31]. On the contrary, a decrease in dry matter intake was observed in adult Holstein cows supplemented with *Saccharomyces cerevisiae* fermentation product after peak lactation [32], although other studies found no effect on voluntary intake [32]. The supplementation of dairy goats with postbiotics yeast fermentation products similar to those used in our study showed no differences in apparent nutrient digestibility, except for fibre digestibility [16]. However, higher dry matter intake and dry matter, protein, and fibre digestibilities were reported in lambs supplemented with postbiotics from *Lactobacillus plantarum* [33]. A meta-analysis of supplementation with postbiotics derived from *Saccharomyces cerevisiae* showed that voluntary intake increased early in lactation, but decreased later, even when supplementation was maintained [34]. In our study, the PR treatment shows a higher voluntary intake than the PC and CT treatments, in parallel with a higher apparent digestibility of nutrients, especially after calving. The higher digestibility may be due to the fact that the higher dry matter intake during ruminal fermentation provides a higher amount of volatile fatty acids and nitrogen to the ruminal microbiome for microbial synthesis [35], which would consequently increase ruminal digestibility. Using Rusitec fermenters, it was observed that yeast hydrolysate increased the concentrations of ammonia nitrogen, propionic acid and butyric acid, and decreased the concentration of acetic acid and the acetic/propionic ratio [36]. These authors also observed an increase in microbial protein synthesis and bacterial diversity. In addition, other authors suggest that yeast metabolites would have effects on the rumen environment, including increasing pH and altering volatile fatty acids concentrations [37], as well as providing soluble growth factors [38] that stimulate the growth of cellulolytic bacteria [39] and consequently increase the extent of ruminal digestion of fibre and the rate of fibre transit improving dry matter intake. Therefore, it can be speculated that the postbiotic added with the PR treatment provides an improvement in the rumen environment, inducing increased nutrient degradability, and resulting in increased voluntary intake.

The concentration of colostral immunoglobulins was higher in the supplemented treatments than in the control, which was reflected in a higher concentration of protein, being positively correlated [40]. In any case, the concentration of immunoglobulins in colostrum was above 50 g IgG/L in all treatments, a concentration above which the colostrum is considered to be of high quality [41]. Calves are mainly protected by passively transferred immunity from colostrum [42]. Thus, a lower concentration of 50 g IgG/L of colostrum exposes the calf to increased susceptibility to disease in the first days of life, with negative consequences in the medium and long term. In monogastric animals, supplementation with prebiotics and probiotics increases the concentration of immunoglobulins in the colostrum of bitches [43,44] and sows [45,46]. In addition, the concentration of IgG in colostrum is influenced by the duration of the supplementation period [47]. To our knowledge, we have not found any studies reporting similar conclusions in ruminants. However, from the results of our work, we can confirm that supplementing cows with postbiotics increases the concentration of immunoglobulins in colostrum. As a result, it can be speculated that calves from cows supplemented with postbiotics during pregnancy may receive higher levels of immunoglobulins when fed their mothers’ colostrum, which would benefit calf health in the first weeks of life.

The highest milk production was observed with the PC treatment in the current study, while the PR did not differ from the control treatment. The different results may be due to the different nature of the postbiotics evaluated. However, variable results can be found in the literature. By supplementing yeast as a probiotic, some authors have found improvements in milk production both in cows [48] and goats [49], while others have found no effect on production in early lactation [50]. Similarly, milk production responses were variable when yeast-derived postbiotics were used. Some studies described increases in milk production [28,51,52], whereas others did not observe any effect [25,53]. The response of dairy cows to probiotic supplementation, and therefore to postbiotics due to their similar mode of action, depends on the stage of lactation, the type of feed and the forage/concentrate ratio [54], as well as the type of probiotic [53]. In our case, both the physiological state of the cows and the rations were the same in all treatments, so the differences observed could be attributed to the different modes of action of the postbiotics evaluated. However, as these were commercial additives, we have no knowledge of the nature of the additives and the possible differences in how they work.

In the current study, the milk fat concentration of cows fed the PC treatment tended to be higher compared to the other treatments and the protein concentration also tended to be higher than the control treatment. These results, along with the higher milk yield observed for PC treatment, led to significantly higher milk, protein and milk fat yields. These results are in agreement with other studies [24,55], which reported that cows supplemented with yeast culture as a probiotic had a higher milk fat content and a higher milk fat yield, although they did not observe a higher milk protein yield. However, other authors found no differences when supplementing with yeast-derived postbiotics [26]. The proportion of protein in the milk of cows supplemented with the PR treatment tended to be higher than in the control and PC treatments. This result, along with a non-significantly higher lactose concentration, caused a higher proportion of solids-not-fat of milk in PR compared to the control and PC treatments. Solids-not-fat of milk consist of proteins (mainly casein), carbohydrates (mainly lactose), minerals, and vitamins that add texture and creaminess to the milk [56], which are highly valued by consumers. Thus, the addition of postbiotic PR to the cows’ rations could improve the organoleptic characteristics of the milk. In all treatments, urea values were always within the range considered optimal (210 and 320 mg/L), indicating that the rations had an optimal protein and energy intake [57].

## 5. Conclusions

The inclusion of postbiotics in dairy cows during the transition period increased the voluntary intake of dry matter, especially in the PR treatment, with higher apparent total tract digestibility of dry matter, organic matter and neutral detergent fibre. Both treatments, including postbiotics, induced an increase in colostral immunoglobulin concentration. Milk production of cows receiving the PC treatment was the highest, with high fat and protein yields and a higher persistence of the production curve throughout the lactation. Thus, the results of the current study suggest that postbiotic supplementation in late gestation and early lactation could be an effective strategy to improve colostrum immune quality and increase milk yield in dairy cows.

## Figures and Tables

**Table 1:** Ingredients and chemical composition of the pre- and postpartum total mixed rations and the concentrate supplied.

	Prepartum	Postpartum	Additional Concentrate ^1^
Diet composition (% on dry matter basis)			
Grass silage	41.16	46.81	
Cereal straw	12.14	6.12	
Concentrate ^1^	46.70	47.07	
Chemical composition (% on DM basis)			
Dry matter (DM)	46.98	43.01	88.71
Organic matter (OM)	90.39	90.26	91.36
Crude protein (CP)	13.10	13.96	22.82
Crude fibre (CF)	25.92	21.29	4.96
Ether extract (EE)	3.60	4.08	3.54
Nitrogen-free extract (NFE)	47.76	50.94	60.04
Starch	13.89	17.40	37.52
Neutral detergent fibre (NDF)	48.80	43.19	20.17
Acid detergent fibre (ADF)	30.63	27.19	8.56
Net energy for lactation (Mcal/kg DM)	1.44	1.52	1.89

^1^ Provided by Sociedad Asturiana de Servicios Agropecuarios, S.L. (Siero, Asturias, Spain). Concentrate ingredients (% as fed): Corn flaked: 38.00; Maize grain: 25.78; Roasted soybean: 20.52; Wheat bran: 9.78; Salts of FA bypass: 2.82; Sodium bicarbonate: 1.40; Calcium carbonate 0.76; Yuca extract: 0.39; Sodium chloride: 0.30; Magnesium oxide: 0.15; Barley grain: 0.10. Additional concentrate ingredients (% as fed): Maize grain: 50.0; Roasted soybean: 35.0; Barley grain: 3.0; Salts of FA bypass: 2.6; Rye grain: 2.0; Soybean hulls: 2.0; Sunflower seed meal: 1.8; Sodium bicarbonate: 1.0; Magnesium oxide: 1.0; Molasses: 1.0; Mineral–vitamin premix: 0.7.

**Table 2:** Average nutrient intake and apparent digestibility of nutrients according to experimental treatments during the prepartum period.

	CT	PC	PR	rsd	*p*
Intake (kg/day)					
Dry matter	10.26 ^b^	10.46 ^ab^	10.97 ^a^	0.498	0.027
Organic matter	9.28 ^b^	9.50 ^ab^	9.94 ^a^	0.451	0.025
Crude protein	1.44 ^b^	1.60 ^a^	1.57 ^a^	0.068	0.001
Neutral detergent fibre	4.37 ^b^	4.71 ^a^	4.72 ^a^	0.233	0.009
Digestibility (%)					
Dry matter	62.00	66.91	64.92	4.030	0.091
Organic matter	62.87	67.59	66.03	3.889	0.088
Crude protein	60.16 ^b^	68.20 ^a^	63.01 ^ab^	4.059	0.005
Neutral detergent fibre	50.38	52.30	52.12	5.532	0.773
Nitrogen balance (%)	45.21	54.64	48.77	7.253	0.344

CT: control; PC: Probisan-Ruminants-C^®^; PR: Probisan-Ruminants^®^; rsd: residual standard deviation; *p*: significance, different letters in the same item indicate statistical differences between treatments.

**Table 3:** Average nutrient intake and apparent digestibility of nutrients according to experimental treatments during the postpartum period.

	CT	PC	PR	rsd	*p*
Intake (kg/day)					
Dry matter	18.37 ^b^	18.06 ^b^	19.88 ^a^	1.118	0.001
Organic matter	16.53 ^b^	16.49 ^b^	17.95 ^a^	1.014	0.015
Crude protein	3.35 ^a^	3.05 ^b^	3.24 ^a^	0.167	0.008
Neutral detergent fibre	5.90 ^b^	6.29 ^b^	7.30 ^a^	0.435	0.001
Digestibility (%)					
Dry matter	64.00 ^b^	64.76 ^b^	73.44 ^a^	7.406	0.036
Organic matter	65.90 ^b^	66.36 ^b^	74.68 ^a^	6.952	0.035
Crude protein	55.02	55.56	63.56	8.921	0.131
Neutral detergent fibre	57.67 ^b^	55.49 ^b^	71.65 ^a^	8.686	0.003
Nitrogen balance (%)	41.28	44.85	50.32	11.040	0.307

CT: control; PC: Probisan-Ruminants-C^®^; PR: Probisan-Ruminants^®^; rsd: residual standard deviation; *p*: significance, different letters in the same item indicate statistical differences between treatments.

## Data Availability

All data used to support the findings of this study are included in the article.
